# *TERT* promoter mutations and monoallelic activation of *TERT* in cancer

**DOI:** 10.1038/oncsis.2015.39

**Published:** 2015-12-14

**Authors:** F W Huang, C M Bielski, M L Rinne, W C Hahn, W R Sellers, F Stegmeier, L A Garraway, G V Kryukov

**Affiliations:** 1 Department of Medical Oncology, Dana-Farber Cancer Institute, Boston, MA, USA; 2 Department of Medicine, Harvard Medical School, Boston, MA, USA; 3 Cancer Program, The Broad Institute of Harvard and MIT, Cambridge, MA, USA; 4 Department of Neurology, Harvard Medical School, Boston, MA, USA; 5 Oncology Disease Area, Novartis Institutes for Biomedical Research, Cambridge, MA, USA; 6 Division of Genetics, Brigham and Women's Hospital, Boston, MA, USA

## Abstract

Here we report that promoter mutations in telomerase (*TERT*), the most common noncoding mutations in cancer, give rise to monoallelic expression of *TERT*. Through deep RNA sequencing, we find that TERT activation in human cancer cell lines can occur in either mono- or biallelic manner. Without exception, hotspot *TERT* promoter mutations lead to the re-expression of only one allele, accounting for approximately half of the observed cases of monoallelic *TERT* expression. Furthermore, we show that monoallelic *TERT* expression is highly prevalent in certain tumor types and widespread across a broad spectrum of cancers. Taken together, these observations provide insights into the mechanisms of *TERT* activation and the ramifications of noncoding mutations in cancer.

## Introduction

In healthy tissues, expression of the telomerase gene (*TERT*) is generally restricted to the germline and stem cells. However, *TERT* activation, necessary for the maintenance of unlimited replicative potential, occurs commonly in many cancers. Recurrent mutations in the *TERT* promoter region were first discovered in melanoma and have since been found to be the most common somatic mutations in many cancers.^1, 2, 3, 4^ Two mutually exclusive cytidine-to-thymidine mutations, C228T and C250T (chr5:1 295 228 C>T and 1 295 250 C>T; hg19), positioned at 124 and 146 base pairs upstream of the ATG translational start site of *TERT*, respectively, are found in ~70% of melanomas, 80–90% of glioblastoma multiforme, 60% of hepatocellular carcinoma, 60% of bladder cancer, 70% of basal cell carcinoma, 50% of cutaneous squamous cell carcinoma and up to 30% of thyroid cancers. Other recurrent but much less frequent mutations in the *TERT* promoter (occurring at positions chr5: 1 295 228 C>A, 1 295 242–1 295 243 CC>TT and 1 295 161A>C) have also been observed.^5^ All of these mutations create putative consensus ETS transcription factor binding sites (GGAA/T) and are hypothesized as a mechanism of *TERT* activation.^1, 2^ Subsequent studies in glioblastoma and bladder cancer demonstrated that *TERT* promoter mutations correlate with higher *TERT* mRNA and protein expression and elevated telomerase activity.^6^ Furthermore, recent data demonstrate that the mutant *TERT* promoter can be bound and activated by GABP, an ETS transcription factor.^7^ The recurrent *TERT* promoter mutations also prevent silencing of *TERT* upon differentiation of stem cells.^8^ For this study, we used whole-genome DNA and deep RNA sequencing data from 329 cancer cell lines representing a wide range of tumor types from the Cancer Cell Line Encyclopedia (CCLE)^9, 10^ to interrogate the *TERT* locus and determine the modes of *TERT* activation.

## Results and discussion

**Figure 1:**
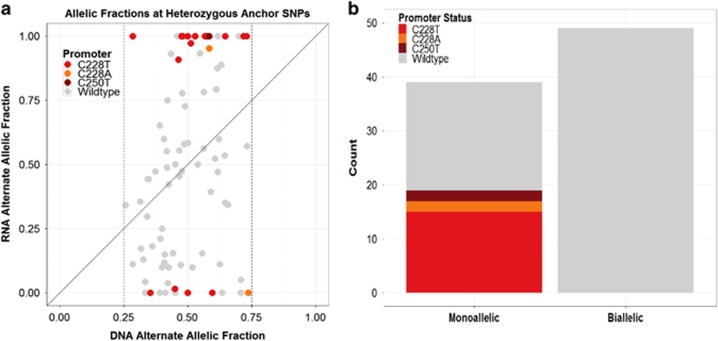
Identification of cancer cell lines with monoallelic *TERT* expression. (**a**) Fraction of sequencing reads harboring nucleotide that differs from reference human genome for heterozygous anchor SNPs. DNA and RNA alternate allelic fractions are plotted along the x axis and y axis, respectively. Analysis was performed on 88 cell lines with a DNA alternate allelic fraction between 0.25 and 0.75 in the DNA sequences of expressed exonic and untranslated regions of the *TERT* gene. Strong deviation from the diagonal indicates allelic bias in expression. Individual points represent samples, and those harboring hotspot promoter mutations are highlighted. Nineteen cell lines harbored one of the hotspot promoter mutations C228T, C228A or C250T and were found within the 88 cell lines that met the criteria for assessment of allelic bias. (**b**) Bar plot of promoter mutation status in cell lines with monoallelic and biallelic expression of the *TERT* gene. *TERT* expression was classified as monoallelic in samples with heterozygous anchor SNPs in the *TERT* gene for which expression of a major allele was >10-fold higher than expression of the minor allele in RNA versus DNA.

To analyze *TERT* expression, we first identified cell lines with heterozygous ‘anchor' single-nucleotide polymorphisms (SNPs) (defined here as having at least three reference and alternate reads with a DNA alternate allelic fraction between 0.25 and 0.75) in the DNA sequences of expressed exonic and untranslated regions of the *TERT* gene. Cell lines with insufficient RNA sequencing coverage of anchor SNPs (i.e., fewer than eight reads) and cell lines with high-level copy-number amplification or deletion of the *TERT* locus were excluded from downstream analyses. In total, 88 CCLE lines met these criteria and had at least one heterozygous SNP that would allow evaluation of an allelic bias in RNA expression (Supplementary Table 1). The number of sequencing reads that supported the reference or alternative alleles were counted both in whole-genome sequencing and RNA-Seq data and used to quantify allelic imbalance (Figure 1a). In total, 39 out of 88 cell lines (44.3%) showed a monoallelic pattern of *TERT* expression with a cutoff of one allele being expressed at least 10-fold higher than the other allele in RNA versus DNA.

We then investigated the relationship between the monoallelic expression pattern and known mechanisms of *TERT* activation. To identify the recurrent *TERT* promoter mutations, we used whole-genome sequencing data from the CCLE to interrogate the *TERT* promoter region in 329 cell lines, of which 316 lines had sufficient coverage at all previously defined hotspots (Supplementary Table 2). In total, 60 lines were found to harbor known *TERT* promoter mutations (Supplementary Table 2 and Supplementary Figure 1). Promoter mutation status was determined by computing allele counts at genomic loci for five known recurrent somatic mutations in the *TERT* gene promoter region (positions 1 295 191, 1 295 228, 1 295 242, 1 295 243 and 1 295 250 in chromosome 5; Supplementary Table 2). Samples in which mutations were detected at any of these positions were classified as promoter-mutant. Consistent with previous reports, all detected hotspot mutations were mutually exclusive.

We hypothesized that the *TERT* promoter mutations would lead to activation of only one allele. Indeed, we found that all of the cell lines harboring *TERT* promoter mutations showed monoallelic expression (Figure 1b). The correlation of monoallelic expression with the known hotspot *TERT* promoter mutations was highly statistically significant (Fisher's exact test, one-tailed; *P*<10^−8^) and overall, nearly half (48.7% 19/39) of the cell lines that showed monoallelic expression could be accounted for by the presence of the recurrent *TERT* promoter mutations (Figure 1b). We did not observe a significant difference in *TERT* mRNA expression levels in *TERT* promoter-mutant-positive cell lines compared with *TERT* promoter wild-type cell lines (Supplementary Figure 2).

**Figure 2:**
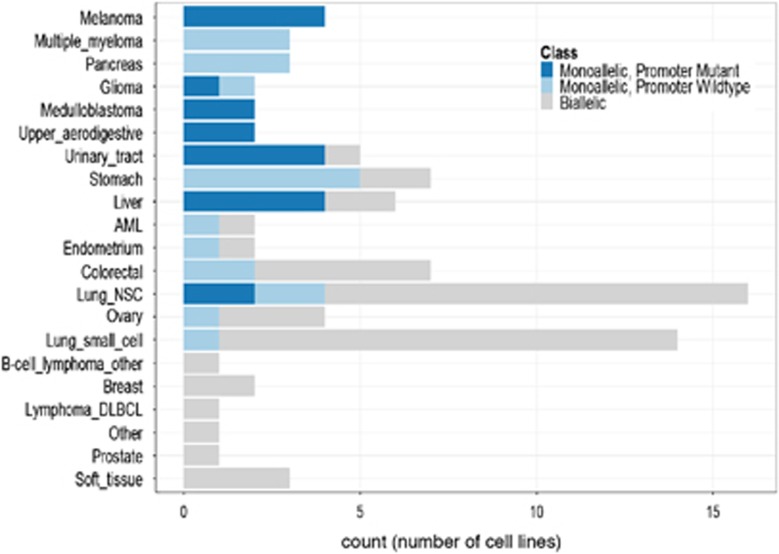
Distribution of cell lines with monoallelic *TERT* expression across tissue lineages. We found a highly significant difference in the distribution of samples with monoallelic *TERT* expression across lineages when compared with cell lines with biallelic *TERT* expression for both promoter-mutant and promoter-wild-type cell lines (*P*=1.59 × 10^−6^ and *P*=0.006, respectively; Fisher–Freeman–Halton test).

We also found that the prevalence of monoallelic expression of *TERT* varied markedly among different cell lineages (Figure 2). Cancer types such as melanoma, multiple myeloma, pancreas, upper aerodigestive tract, urinary tract and stomach almost exclusively showed monoallelic expression of *TERT*. Overall, in over 70% of lineages, at least one cell line showed evidence of monoallelic activation of *TERT*. Among cell lines with monoallelic *TERT* expression, we found a highly significant difference in the distribution across lineages when compared with cell lines with biallelic expression of *TERT* for both promoter-mutant and promoter-wild-type cell lines (*P*=1.59 × 10^−6^ and *P*=0.006, respectively; Fisher–Freeman–Halton test; Figure 2). We did not observe significant differences in mRNA expression levels between cell lines that displayed monoallelic expression or biallelic expression of *TERT*. These data suggest that somatic monoallelic activation of *TERT* may show evidence of a transcriptional compensation phenomenon that has been observed in monoallelic expression in other models (Supplementary Figure 3).^11^

**Figure 3:**
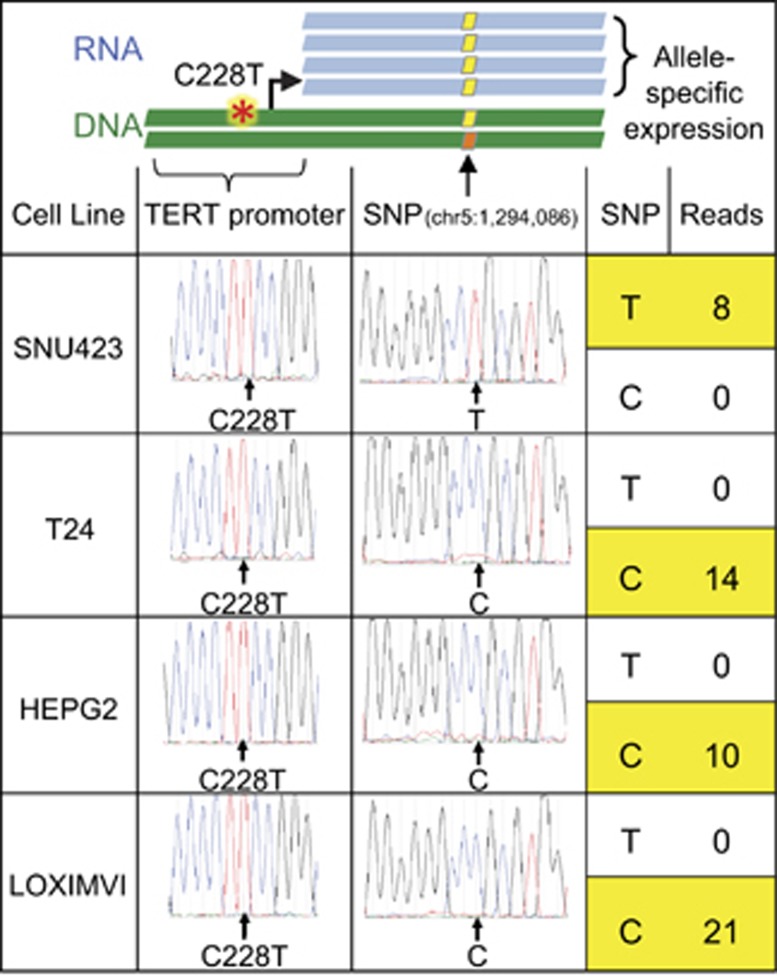
Relationship between *TERT* promoter mutations and allele-specific expression. DNA was isolated from cell lines with a *TERT* promoter mutation and nearby heterozygous exon 2 anchor SNP (first column) using the DNeasy Blood and Tissue Kit (Qiagen, Hilden, Germany). The genomic region containing the *TERT* promoter and anchor SNP was PCR amplified, gel purified and cloned using the Zero Blunt PCR Cloning Kit (Life Technologies, Carlsbad, CA, USA). Sanger sequencing chromatograms of individual clones demonstrate the *TERT* promoter mutation (second column) in *cis* with one of the alleles at the anchor SNP (third column). RNA sequencing confirmed allele-specific expression by identifying exclusive expression of the SNP that was in *cis* with the *TERT* promoter mutation (fourth column).

To test whether the promoter mutations occurred in *cis* with the alleles, which showed monoallelic expression, we subcloned the region of the *TERT* gene encompassing the heterozygous ‘anchor' SNP (rs2736098) at position chr5:1 294 086 and the promoter mutation C228T (chr5:1 295 228 C>T) in four unrelated cell lines. Using Sanger sequencing, we found that the allele that showed monoallelic expression at this position was in *cis* with the mutant promoter allele and not the wild-type promoter allele in all lines tested (*P*=0.05; Student's *T*-test; Figure 3).

Although all cell lines with *TERT* promoter mutations showed a monoallelic pattern of *TERT* expression, these cell lines accounted for only half of the samples that exhibited strong allelic bias. Monoallelic expression in the remaining samples could be potentially explained by two major mechanisms—unidentified *cis*-acting genetic alterations that affect only one chromosome, or mitotically stable epigenetic activation of one allele. To investigate the first possibility, we analyzed the 10 kb region upstream of the *TERT* gene. We failed to detect a significant enrichment in proximal *cis* events (point mutations or rearrangements) in lines with an unexplained monoallelic pattern of *TERT* activation as compared with lines with biallelic expression of *TERT* (Supplementary Figure 4 and Supplementary Data). These data suggest that other proximal *cis*-acting genetic alterations do not appear to explain monoallelic expression of *TERT* in the cancer cell lines we analyzed that do not harbor a hotspot mutation in the *TERT* promoter. However, we cannot rule out the possibility that some of the substitutions found within the promoter or that genomic rearrangements beyond the upstream 10 kb could potentially contribute to monoallelic expression.

Activation of *TERT* expression is a crucial step in the progression of many cancers and understanding the molecular mechanisms of such activation is important for the understanding of tumorigenesis. Recent genome-wide analyses have highlighted that noncoding mutations in the *TERT* promoter are among the most common mutations in human cancer.^12, 13, 14^ We observed that the monoallelic mode of *TERT* activation is widespread, with half of cases explained by the known promoter mutations and certain lineages showing almost exclusive monoallelic expression of *TERT*. These observations highlight the heterogeneity of molecular mechanisms to activate *TERT*, some of which are still to be discovered. Here we show that the integration of DNA and RNA sequencing to determine monoallelic expression can be used to identify and characterize *cis*-acting noncoding mutations in cancer. While our results establish *TERT* monoallelic expression in cell lines across many tumor types, confirmation of these results in primary tumors will be needed. Whether other mechanisms such as random monoallelic expression or epigenetic mechanisms account for the non-promoter-mutant-associated *TERT* monoallelic expression remains an active area of investigation.^15, 16, 17^ Aside from *TERT*, recent studies demonstrate that other promoter regions may be recurrently mutated in cancer.^18, 19, 20^ Our findings suggest that somatic monoallelic activation of *TERT* is a common mechanism of *TERT* expression and that *TERT* promoter mutations drive monoallelic expression of *TERT*.

## Notes

Drs Sellers and Stegmeier are employees of Novartis. Dr Garraway has received research funding form Novartis and holds equity in Foundation Medicine. The other authors declare no conflict of interest.
