# Supplementary figures and images for: TERT promoter mutations and monoallelic activation of TERT in cancer
Source: Oncogenesis. 2015 Dec 14;4(12):e176–. doi: 10.1038/oncsis.2015.39 (PMC4688396; doi:10.1038/oncsis.2015.39)

Supp Fig 1

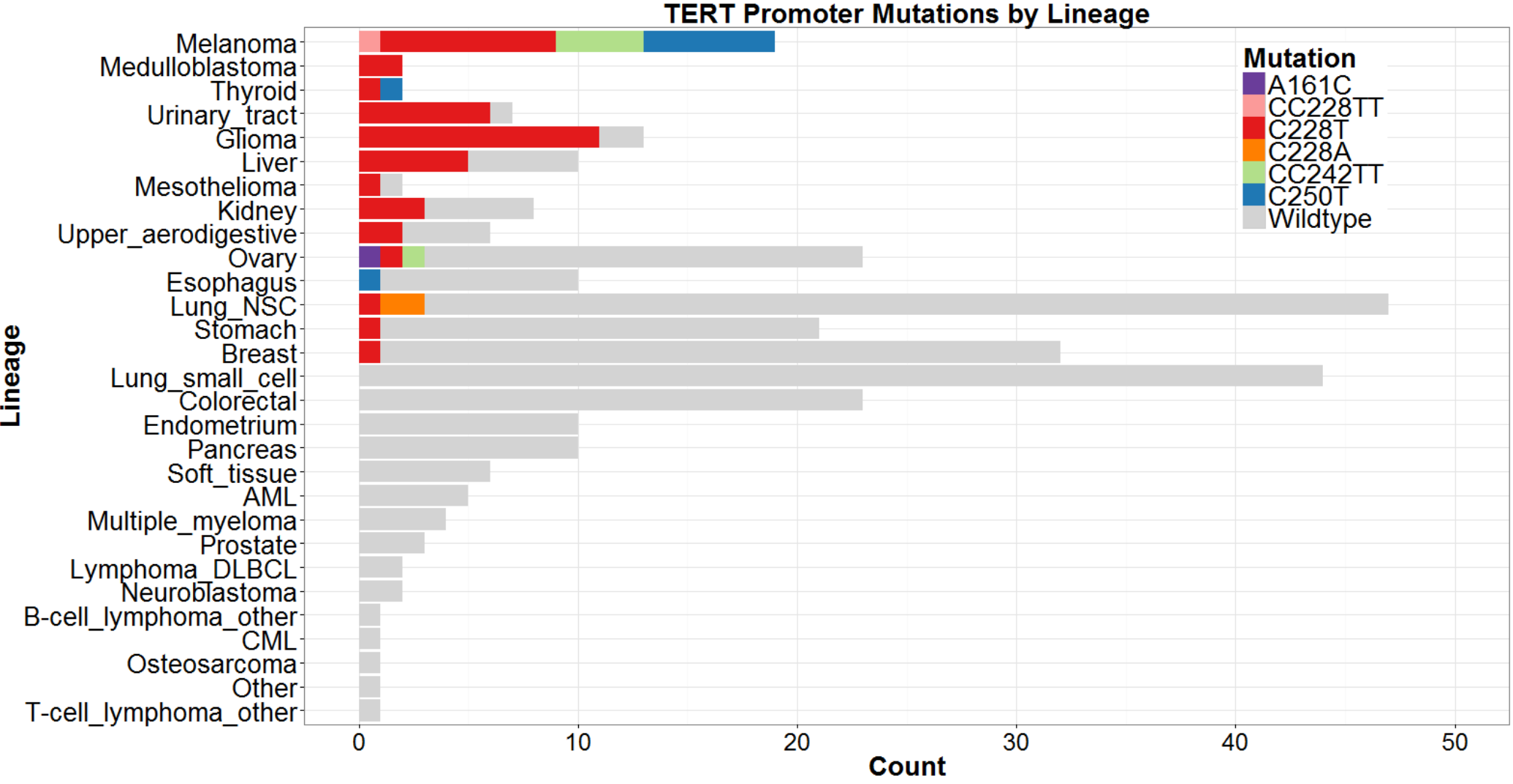

Supplement: Supplementary Figure 1 [file oncsis201539x1.pdf]

Supp Fig 2

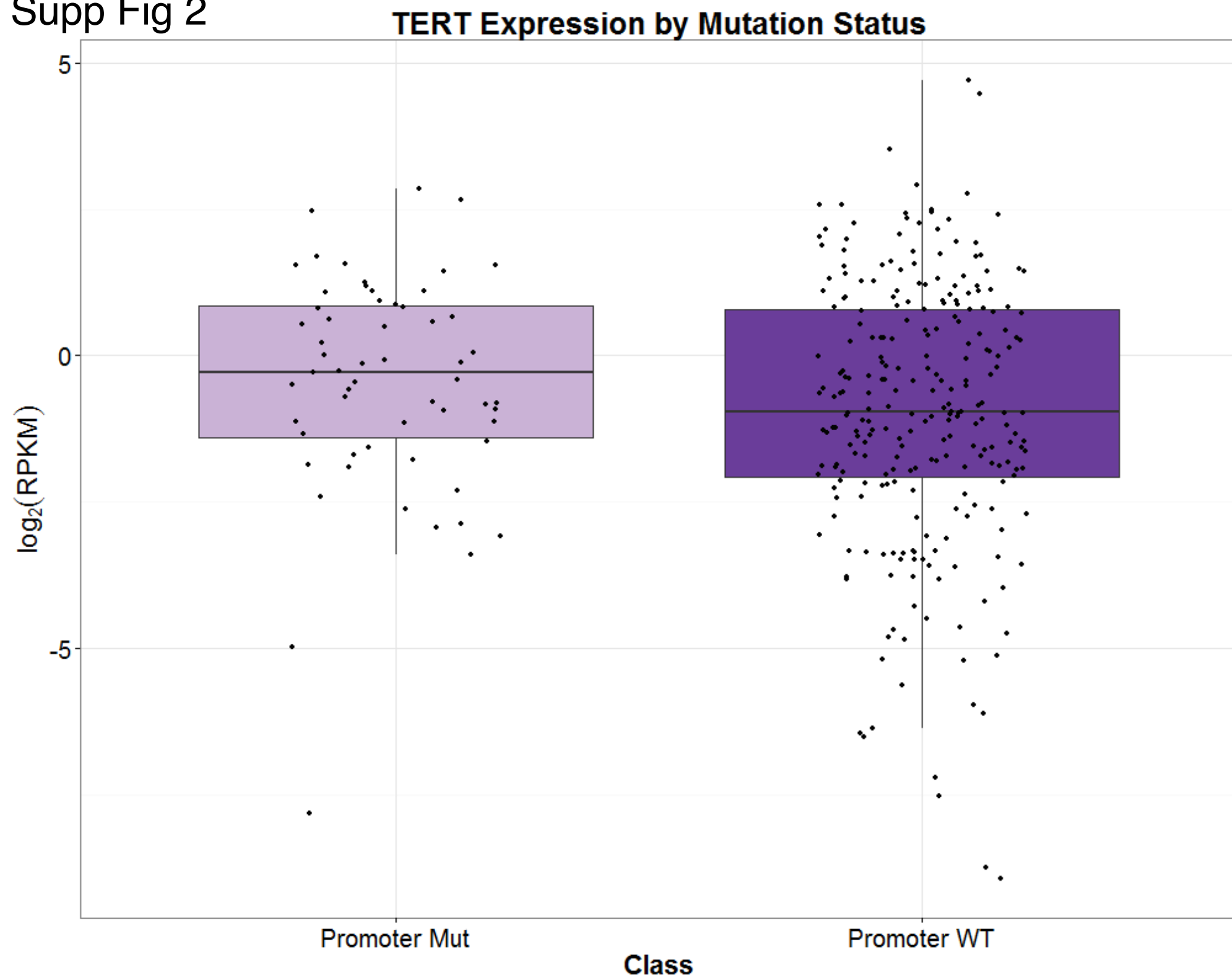

Supplement: Supplementary Figure 2 [file oncsis201539x2.pdf]

Supp Fig 3

TERT Expression by Class

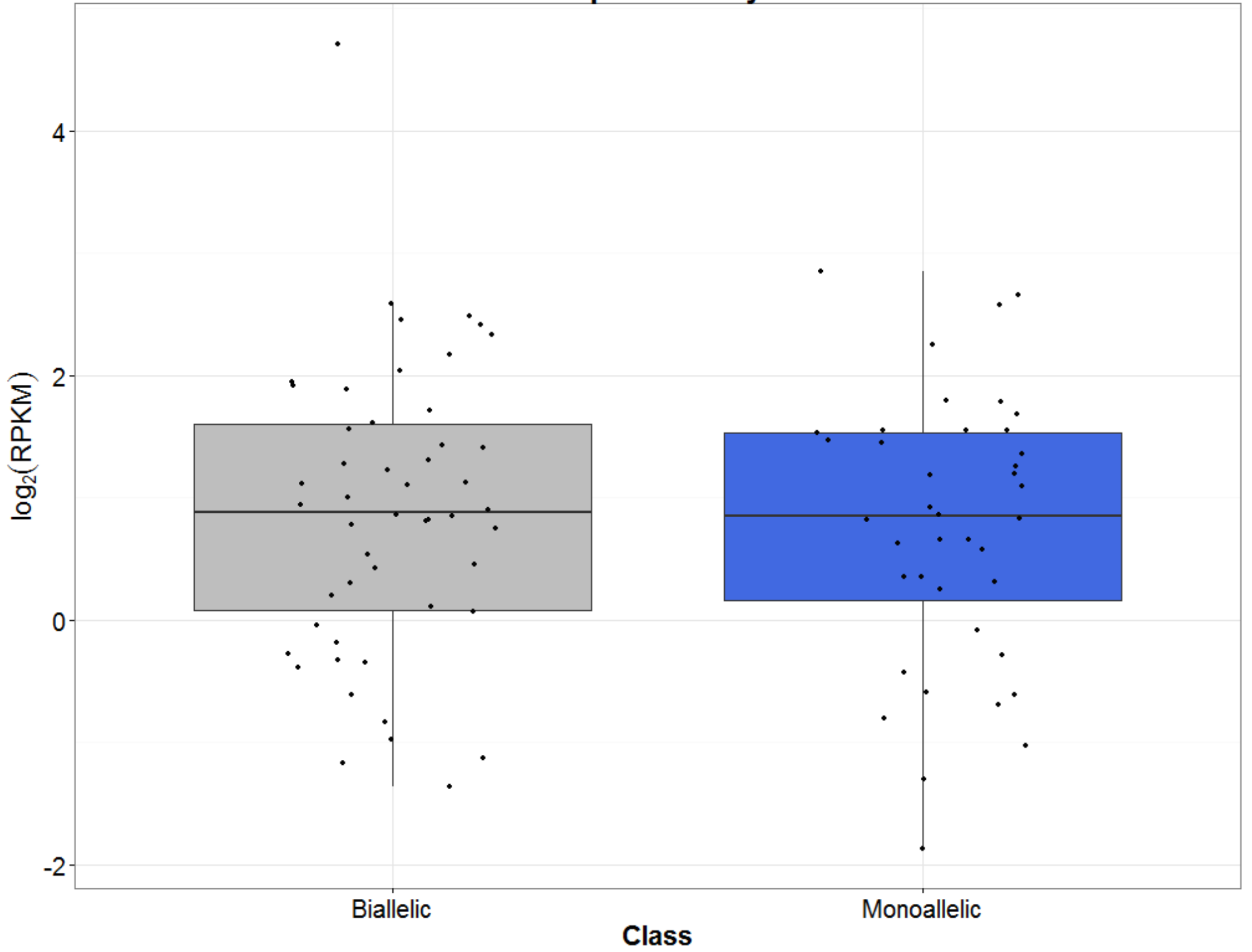

Supplement: Supplementary Figure 3 [file oncsis201539x3.pdf]

Supp Fig 4

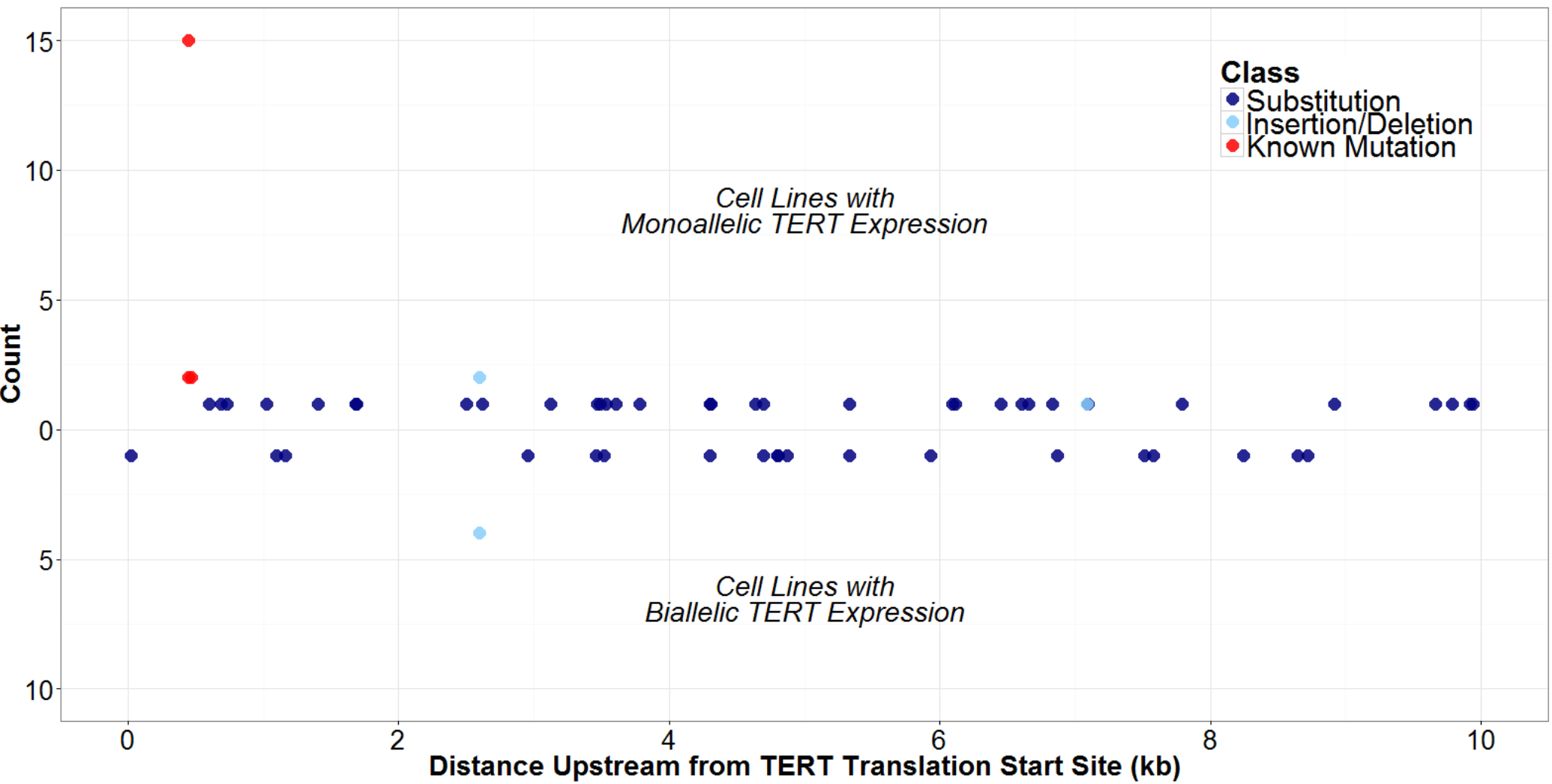

Supplement: Supplementary Figure 4 [file oncsis201539x4.pdf]
