# Supplementary material for: TERT promoter mutations and monoallelic activation of TERT in cancer
Source: Oncogenesis. 2015 Dec 14;4(12):e176–. doi: 10.1038/oncsis.2015.39 (PMC4688396; doi:10.1038/oncsis.2015.39)
Supplement: Supplementary Figure Legends [file oncsis201539x5.docx]

**Supplemental Figure Legends**

**Supplemental Figure 1.** Bar plot of number of cell lines harboring recurrent TERT promoter mutations across 329 cell lines from the CCLE categorized by tissue type origin.

**Supplemental Figure 2.** TERT expression in the subset of 316 samples for which we were able to classify TERT promoter mutation status at known recurrent events in the promoter region. Individual points represent samples, and expression is given in terms of log_2_ RPKM (reads per kilobase per million mapped reads).

**Supplemental Figure 3.** TERT expression in the subset of 88 samples for which we were able to identify monoallelic or biallelic expression patterns using heterozygous anchor SNPs. Individual points represent samples, and expression is given in terms of log_2_ RPKM (reads per kilobase per million mapped reads).

**Supplemental Figure 4:** Landscape of nucleotide substitutions, short insertions/deletions, and known hotspot mutations in 88 CCLE cell lines in CCLE for the region 10kb upstream of the TERT translation start site. Known hotspot promoter mutations are highlighted. Observed counts are depicted for samples with monoallelic and biallelic TERT expression.
