# Supplementary material for: TERT promoter mutations and monoallelic activation of TERT in cancer
Source: Oncogenesis. 2015 Dec 14;4(12):e176–. doi: 10.1038/oncsis.2015.39 (PMC4688396; doi:10.1038/oncsis.2015.39)
Supplement: Supplementary Table Legends [file oncsis201539x8.docx]

**Supplemental Table 1**

Evidence for an allelic bias in expression. Read counts from DNA and RNA sequencing which support reference and alternative alleles are listed for all heterozygous anchor SNPs found in our final set of cell lines used for analysis. P-values are show for the Fisher-test used to test whether the relative proportions of read counts are independent of data source (i.e., DNA vs. RNA). Monoallelic expression calls are based on the normalized allelic ratio, defined as:

$$\frac{(RNA read counts for major allele in RNA / RNA read counts for minor RNA allele in RNA)}{(DNA read counts for major allele in RNA / DNA read counts for minor RNA allele in RNA)}$$

Allelic ratios greater than 10 were classified as monoallelic expression. Finally, TERT promoter status is indicated for all cell lines.

**Supplemental Table 2**

Cell lines TERT promoter status
