# Supplementary material for: TERT promoter mutations and monoallelic activation of TERT in cancer
Source: Oncogenesis. 2015 Dec 14;4(12):e176–. doi: 10.1038/oncsis.2015.39 (PMC4688396; doi:10.1038/oncsis.2015.39)
Supplement: Supplementary Methods [file oncsis201539x9.docx]

**Supplemental Methods**

*Whole Genome Sequencing: library construction, sequencing and alignment*

All whole genome sequencing data used herein were previously described.^3^

*RNA-Seq: library construction, sequencing and alignment*

RNA-Non strand-specific RNA sequencing was performed using large-scale, automated variant of the Illumina Tru Seq™ RNA Sample Preparation protocol. Oligo dT beads were used to select polyadenylated mRNA. Selected RNA was then heat fragmented and randomly primed before cDNA synthesis. Fragment insert size was set to 400nt to maximize power to detect fusions. The resultant cDNA was prepared using Illumina library protocols (end repair, base ‘A’ addition, adapter ligation, and enrichment) using Broad designed indexed adapters for multiplexing. Sequencing was performed on the Illumina HiSeq 2000 or HiSeq 2500 instruments, with sequence coverage per sample of no less than 100 million paired reads of length 101 nucleotides. Reads were aligned to the human reference genome build hg19 using TopHat version 1.4.

*Mutation calling*

Mutations were called from whole genome sequences using the MuTect algorithm (Cibulskis et al, *Nat Biotechnol.* 2013) in the “no matching normal” mode.^[[1]](#endnote-1)^ For all sites at which differences from the human reference genome (hg19) were detected in WGS data, read counts for reference and alternative alleles were determined in RNA-Seq.

*Evaluating TERT promoter mutation status for known recurrent somatic nucleotide substitutions*

Allele counts at genomic loci for five known recurrent somatic mutations in the TERT gene promoter region (positions 1295191, 1295228, 1295242, 1295243 and 1295250 in chromosome 5) were determined using MuTect in the “force call” mode. Samples in which mutations were detected at these positions were classified as harboring known recurrent events. No samples were found to harbor more than one known recurrent nucleotide substitution in the *TERT* promoter region.

Samples were classified as wild type for a given known recurrent event in the TERT promoter region if at least 9 reads supporting the wild type allele were present with no alternate reads supporting a mutation. Indeed, samples were classified as wild type for all known recurrent *TERT* promoter mutations only if all five loci had sufficient coverage to confidently call them as wild types individually.

*Heterozygous anchor SNP selection for allele-specific expression analysis*

For all samples, sequencing reads supporting reference or alternative alleles were counted in both WGS and RNA-Seq data. SNPs with fewer than 3 reference or alternative reads in DNA or insufficient coverage (less than 12 reads for DNA and less than 8 for RNA) were excluded from downstream analysis. Intronic SNPs and SNPs that exhibited strong allelic imbalance at the DNA level with a dominant allele representing more than 75% of total reads were also excluded from consideration. In total, 90 samples had at least one acceptable anchor heterozygous SNP that could be used to determine the presence or absence of allele-specificity of TERT expression. Of these, two samples were excluded on the basis of insufficient coverage at one or more recurrent somatic mutation loci. In cases where more than one acceptable anchor SNP was present, the SNP with the highest RNA coverage was used.

*Detection of allele-specific expression*

In samples with heterozygous anchor SNPs in the *TERT* gene for which expression of a major allele was more than 10-fold higher than expression of the minor allele, *TERT* expression was classified as being monoallelic.

*TERT gene expression*

Gene-level *TERT* expression was quantified from RNA-Seq data using a computational pipeline developed for the GTEx project [The GTEx Consortium, Science, 2015]

*Determination of relationship between allele-specific expression and TERT promoter mutation*

Cancer cell lines exhibiting mono-allelic TERT expression that contained a TERT promoter mutation along with a nearby heterozygous anchor SNP at 1,294,086 were identified.  Four of these cell lines (T24, SNU423, HEPG2, and LOXIMVI) were obtained from the Biological Samples Platform at the Broad Institute and genomic DNA was isolated from each of these cell lines using the DNeasy Blood and Tissue Kit (Qiagen).  PCR was performed to amplify a genomic region containing both the anchor SNP and the *TERT* promoter mutation using PCR primers upstream of the *TERT* promoter (5'-CACAGCCTAGGCCGATTC-3') and downstream of the anchor SNP (5'-CAGCTGCTCCTTGTCGC-3') using the protocol previously described.  PCR products were gel purified using the Qiaquick Gel Extraction kit (Qiagen) and ligated into the pCR-Blunt vector using the Zero Blunt PCR Cloning Kit (Life Technologies).  Stbl3 competent bacteria (Life Technologies) were transformed and plasmid DNA was isolated from individual bacterial colonies using the Qiaprep Spin Miniprep Kit (Qiagen).  Sanger sequencing of individual DNA clones was performed to determine whether the 1,294,086 anchor SNP was in *cis* with the *TERT* promoter mutation.

1. Cibulskis K, et al. [Sensitive detection of somatic point mutations in impure and heterogeneous cancer samples.](http://www.ncbi.nlm.nih.gov/pubmed/23396013) *Nat Biotechnol.* 2013 Mar;31(3):213-9. [↑](#endnote-ref-1)
